# Randomized controlled trials of endovascular therapy for acute ischemic stroke with medium or distal vessel occlusion: a study level metaanalysis

**DOI:** 10.1186/s42466-025-00400-4

**Published:** 2025-06-26

**Authors:** Peter D. Schellinger, Georgios Tsivgoulis, Benedikt Frank, Thomas Liebig, Martin Köhrmann

**Affiliations:** 1 Department of Neurology and Neurogeriatry, John Wesling Medical Center Minden, UK RUB University Clinic of the Ruhr-Universität Bochum, Hans-Nolte-Str.1, 32429 Minden, Germany; 2 Second Department of Neurology, School of Medicine, “Attikon” University Hospital, National and Kapodistrian University of Athens, Athens, Greece; 3 Department of Neurology, Medizinisches Zentrum, University Clinic at Essen, Hufelandstraße 55, 45147 Essen, Germany; 4 Institut für Diagnostische und Interventionelle Neuroradiologie, LMU-Universitätsklinikum München, Marchioninistrasse 15, 81377 Munich, Germany

## Abstract

**Introduction:**

A decade ago, endovascular therapy (EVT) for acute ischemic stroke (AIS) with large vessel occlusion (LVO) has been established as standard of care. It is still a matter of debate whether EVT is better and safe for patients with more distal occlusions (DMVO). Three randomized controlled trials investigated the role of EVT on top of best medical treatment (BMT) for patients with DMVO.

**Methods:**

In a narrative review we present the results of 3 randomized controlled trials (RCT), (DISTAL, ESCAPE MeVO, DISCOUNT) of EVT plus BMT versus BMT alone. In addition, we performed a study level meta-analysis with a random-effects model for three endpoints: independent outcome, symptomatic intracranial hemorrhage (sICH) and death.

**Results:**

There was neither a significant effect of EVT plus BMT versus BMT alone on functional outcome (RR 0.92, 95% CI 0.80–1.06, *p* = 0.272), nor did the odds of death differ (OR 1.23, 95% CI 0.76–1.99, *p* = 0.409). The odds for sICH were more than twice as high with EVT (OR 2.38, 95% CI 1.35–4.20, *p* = 0.003).

**Conclusion:**

At present EVT for medium and distal vessel occlusions in AIS patients is not a standard of care. With equipoise for EVT in DMVO now an unbiased and rapid randomization into new and differently designed RCT should be a top priority.

## Introduction

Since publication of the pivotal endovascular therapy (EVT) for large vessel occlusion (LVO) trials a decade ago, EVT for acute ischemic stroke (AIS) has been established as standard of care. It has to be mentioned though, that all of these trials had more or less in common that the targeted patient group had proximal vessel occlusion (mainly ICA and/or MCA-main stem) with high National Institutes of Health Stroke Scale (NIHSS) scores ranging around 17 points and the majority around 85% were pretreated with intravenous thrombolysis (IVT) [1–3]. A major point of debate (also in the joint ESO/ESMINT guidelines [4]) is the effect of EVT in occlusions of the M2 segment or distal thereof. Some of the LVO trials did and others did not allow recruitment of these patients, thus only 8%, a total of just 130 patients had M2 occlusions, albeit proximal or dominant sites with NIHSS scores equal to or higher than 10. In these patients the non-significant odds ratio (OR) in favour of EVT was 1.68 (95% CI 0.9–3.14). The expert consensus based on secondary endpoints and safety (no symptomatic intracranial hemorrhage (sICH)) was that „data is insufficient to give a specific evidence-based recommendation for or against MT plus BMM in patients with M2 occlusions, especially as some patients probably were misclassified as M1 occlusions and then adjudicated as proximal M2 occlusions” [5].

More than 20% of AIS are caused by visualized occlusions in the more distal vasculature (M2–M4) of the middle cerebral artery but also anterior and posterior cerebral arteries. These occlusions are sometimes coined as MeVO/DiVO for medium or distal vessel occlusion. These distal and medium vessel occlusions (DMVO) with NIHSS scores typically below 10 have a clinical course that is not naturally benign with approximately 50% of patients not achieving an excellent functional outcome and one third being dependent or dead. Even with IVT, reperfusion rates are below 50%. During the last years with the development of smaller devices and newer interventional techniques, EVT-treatment has become more common in routine clinical practice in these patients [6, 7]. Yet, aside from an ever-increasing number of reports of observational studies until recently there was a lack of evidence for efficacy and safety of this approach from randomized controlled clinical trials. Therefore, it remains unclear whether the more complex interventional approach combined with the smaller expected effect size in terms of tissue saved and clinical benefit in DMVO patients translates into therapeutic efficacy for EVT on top of best medical treatment (BMT) including IVT.

At the recent international stroke conference (ISC 2025 in Los Angeles, USA), 3 new randomized trials for EVT in DMVO patients have been presented (DISTAL, ESCAPE-MeVO, DISCOUNT) the first two of which were simultaneously published [8, 9]. We aim to present these trials in brief and discuss their implication for current therapeutic strategy and further research. 

## Methods

We performed a study level meta-analysis including the results of the two published (DISTAL, ESCAPE MeVO) and the one presented (DISCOUNT) randomized controlled trials. Three endpoints (functional independence: modified Rankin Scale (mRS) score 0–2, sICH, and death) were chosen for analysis. Data for excellent outcome (mRS 0–1) could not be extracted from the oral presentation of DISCOUNT. Also, for ESCAPE MeVO the sICH population is reported as treated, and for DISCOUNT sICH and death were presented only as treated, intention to treat was not available. We decided to leave the trials in the presented metaanalysis as there were only two cross-overs in ESCAPE-MeVO and there was no difference in the ITT and the as-treated population in the sICH and death rate. In DISCOUNT there were 17 cross-overs randomized to EVT but receiving BMT only, 15 of these because of clinical improvement, but there were no crossovers vice versa. In terms of safety outcomes however, the authors feel that the true attribution of sICH and death to treatment arms yields appropriate results for cautious interpretation.

All crude estimates were pooled using a random-effects model according to DerSimonian and Laird. Although statistical heterogeneity was assessed using the Cochran Q and I^2^ statistics, a random-effects approach was chosen a priori to account for potential clinical and methodological differences between studies, and because the small number of included trials limited the power to detect true heterogeneity. Between-study variance was quantified using Tau^2^. For the qualitative interpretation of heterogeneity, I^2^ values of ≥ 50% were considered indicative of substantial heterogeneity, and values ≥ 75% indicated considerable heterogeneity. Relative risks (RRs) were used for the functional outcome analysis, and ORs were used for the binary safety outcomes (sICH and death), as person-time data were not available.

To test for a statistically significant overall treatment effect in the random-effects model, a chi-squared test with 1 degree of freedom was applied to the squared standardized pooled effect estimate. This test evaluates whether the pooled relative risk differs significantly from 1, and corresponds mathematically to the squared z-statistic derived from the log-transformed effect estimate and its standard error. The chi-squared approach was preferred over the standard normal approximation (z-test) due to the small number of included studies, as it provides a more conservative inference and helps reduce the risk of type I error.

Small-study effects, as a surrogate indicator of publication bias, were evaluated graphically using funnel plots of the unadjusted and adjusted probabilities of independent functional outcome between the two groups. All analyses were performed using StatsDirect version 4.0.4.

## Trials and study level metaanalysis

All 3 trials have commonities but also differences in trial design, devices used, territories included, which makes a direct comparison difficult. DISCOUNT was conducted in France, DISTAL mainly in Europe and ESCAPE MeVO in Canada/USA with few centers in Germany and UK. ESCAPE MeVO is the only of the three trials that included proximal M2 occlusions (approximately 25% of patients, N = 122) without specifying whether that was proximal dominant, codominant or non-dominant. DISTAL included co/-non-dominant M2, M3, M4, A1-A3, P1-P3 segments, DISCOUNT as well but no M4 segments and ESCAPE MeVO no M4, A1 and P1 segments. The NIHSS score at baseline was 5 or more for DISCOUNT, 4 or more or disabling symptoms for DISTAL, and 6 or more or 3–5 with disabling symptoms for ESCAPE MeVO. Time from symptom onset was 6 h or less for DISCOUNT and DISTAL, although DISTAL allowed recruitment up to 24 h when a mismatch on advanced CT/MR imaging was shown. ESCAPE MeVO recruited up to 12 h. DISCOUNT used 90 days dichotomized mRS 0–2 versus 3–6 as a clinical endpoint, the other two ordinal 90 days mRS. DISTAL allowed all devices at the discretion of the interventionalist, ESCAPE MeVO had to use Solitaire X^®^ (Medtronic) as first pass device and in DISCOUNT all others were used but Solitaire X^®^.

A brief overview is given in Table 1 and time metrics are presented in Table 2.

**Table 1:** Trial overview

Trial name	DISCOUNT	DISTAL	ESCAPE MeVO
Full title	Evaluation of Mechanical Thrombectomy in Acute Ischemic Stroke Related to a Distal Arterial Occlusion	EnDovascular Therapy Plus Best Medical Treatment (BMT) versus BMT Alone for MedIum VeSsel Occlusion STroke	EndovaSCular TreAtment to ImProve outcomEs for MEdium Vessel Occlusions
Sample size	161 (of 488 planned)	543	529
Countries	France	Belgium, Finland, Germany, Spain, Switzerland, UK	Canada, Germany, UK, USA
Eligible occlusion location	Distal M2-M3, A1-A3, P1-P3	Co-Non dominant M2-M4, A1-A3, P1-P3	M2-M3, A2-A3, P2-P3
Other imaging criteria	No tandem occlusion	CTP or DWI/FLAIR mismatch 6–24 h	ASPECTS > 7, CT/MR mismatch
Age	≥ 18	≥ 18	≥ 18
NIHSS score	≥ 5	≥ 4 or disabling symptoms	> 5 o3 3–5 with disabling symptoms
Time from SO to randomization	≤ 6 h	≤ 6 h or 6–24 h with mismatch	< 12 h
Primary outcome	mRS 0–2 90 days	90 days mRS	90 days mRS
Allowed revascularization devices	Trevo, Catchview mini, preset Lite, tigertriever 13, max and q aspiration devices	All CE certified devices	Solitaire X^®^
Start date	September 2021	December 2021	April 2022

**Table 2:** Time metrics for EVT + BMT patients

Trial name	DISCOUNT	DISTAL	ESCAPE MeVO
Symptoms to EVT	< 8 h in N = 61 (95%)	4.9 h (2.9–10.7)	N/A
Randomization to EVT (med(IQR); min/max	0.7 h (0.5; 0.9) 0.1 h/3.4 h	36.6 min (24.6–51.9)	N/A
Onset to randomization; med (IWR)	N/A	3.8 h (2.3–9.0)	270 min (160–438)
Door to groin; med(IQR)	N/A	86.5 min (67–112)	95 min (69–136)
Duration EVT (h), med(IQR), min/max	0.8 (0.4; 1.2) 0.1/2.7	N/A	N/A
Door to final angiogram (min); med(IQR)	N/A	N/A	N/A
Total number of passes; med(IQR), min/max	1 (1; 2) 0/7	1 (1; 2)	N/A

DISTAL [8] recruited 543 patients (271 EVT + BMT vs 272 BMT alone) with a mean age of 77 years and median NIHSS score of 6, 65.4% of patients received IVT. 70.9% of patients had occlusions in the MCA territory (44% M2), 18.9% in the P1/P2 territory. The primary outcome was not different in between treatment arms (common OR 0.90 (95% confidence interval, 0.67–1.22; *p *= 0.50). Mortality did not differ significantly (15.5% EVT vs 14% BMT), sICH were numerically higher for EVT but not significantly different between treatment arms (5.9% vs 2.6%). Subgroup analyses for age, NIHSS, occlusion type were irremarkable with only a visual trend shifting from BMT to EVT with increasing NIHSS scores. Excellent outcomes (mRS 0–1) were achieved by 37.6% in the BMT arm and 34.7% in the EVT arm [8].

ESCAPE MeVO [9] included 529 patients (255 EVT + BMT vs 274 BMT alone) with mostly MCA territory occlusions (84.7%). The median age was 75 years and the median NIHSS score was 8, 58% received IVT. The primary endpoint had to be switched as prespecified from an ordinal analysis to the first secondary endpoint, a dichotomous analysis of the mRS (0–1 vs 2–6) because of a violation of the proportional-odds assumption. 41.6% (EVT) versus 43.1% (BMT) achieved a day 90 mRS score of 0 or 1 (adjusted rate ratio, 0.95; 95% CI 0.79–1.15; *p* = 0.61). Independence (mRS 0–2 was achieved by 54.1% (EVT) versus 58.8% (BMT) of patients. There was a significant difference in mortality in favor of BMT (8.4%) versus EVT (13.3%) (adjusted hazard ratio for EVT vs BMT 1.82; 95% CI, 1.06–3.12). Symptomatic ICH occurred in 5.4% in the EVT group and in 2.2% in the BMT group and subarachnoid hemorrhage was ten times more frequent with EVT (19 vs 2 patients, 7.4% vs 0.7%) [9].

DISCOUNT was only orally presented (Clarencon et al., ISC 2025, LA, USA). This trial was terminated prematurely on note of the data safety monitoring board because of futility and safety concerns after 161 patients had been recruited into the trial. The mean age was 74 years, median NIHSS score was 8, and 71% of patients received IVT. The primary endpoint was independence (day 90 mRS 0–2 vs 3–6). 60% in the EVT arm versus 77% in the BMT arm achieved a 90 days mRS of 0–2 (per protocol analysis OR 0.3, 95% CI 0.12–0.74), *p* = 0.009). Numerically there were twice as many sICH (12% vs 6%) but less deaths (3% vs 7%) in the EVT arm.

Preliminary study level metaanalyses presented at the conference from the DISTAL study group (Fischer et al.) showed a trend towards improved outcomes for independence (RR common effects model 0.94, CI 0.86–1.04), and for death (RR 0.78, 95% CI 0.57–1.08) with BMT over EVT. Conversely, there was a significant more than twofold increase of sICH with EVT (RR 2.28, CI 1.33–3.93).

**Fig. 1:**
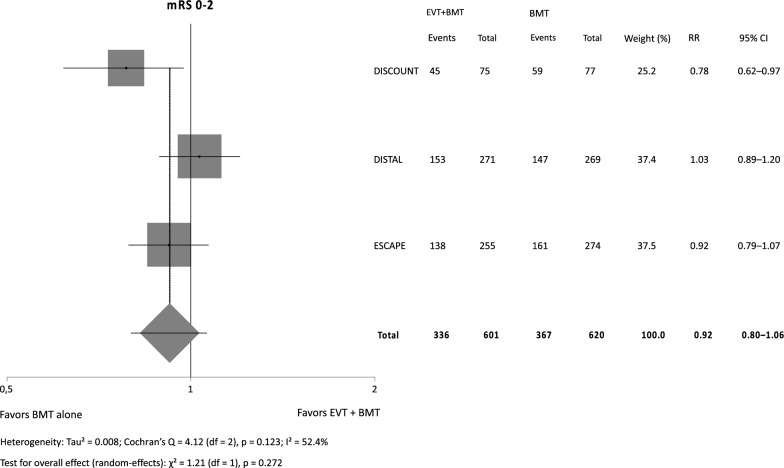
Independent outcome (mRS 0–2)

**Fig. 2:**
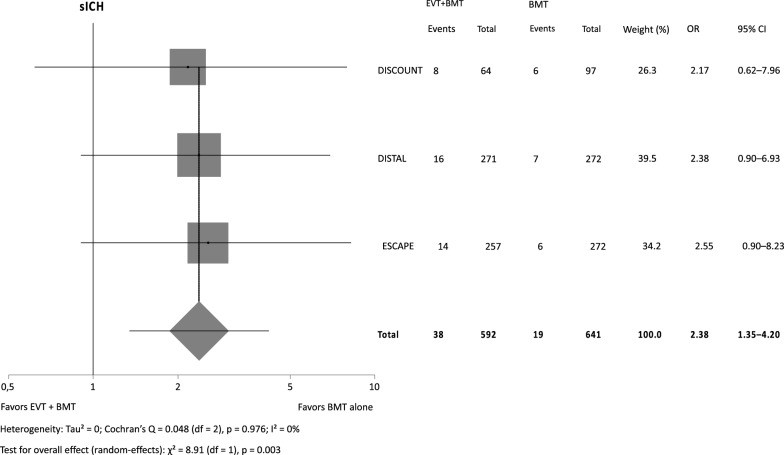
Symptomatic intracranial hemorrhage (sICH), as treated for DISCOUNT and ESCAPE MeVO

**Fig. 3:**
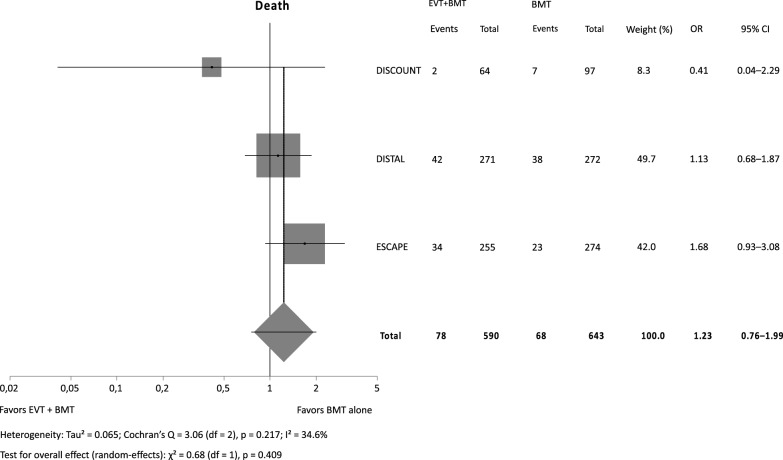
Death, as treated for DISCOUNT

Our metaanalysis (Figs. 1, 2, 3) included a total of 1221 patients (functional outcome) and 1233 patients (sICH and mortality), respectively. For the safety outcomes in DISCOUNT and ESCAPE MeVO only as treated and not intention to treat data were available. There was no significant effect of BMT plus EVT on functional outcome while the point estimate was shifted in favor of BMT alone (RR 0.92, 95% CI 0.80–1.06, *p* = 0.272) with substantial heterogeneity (I^2^ = 52.4%) in between trials. The odds for sICH were significantly increased with EVT (OR 2.38, 95% CI 1.35–4.20, *p* = 0.003) with no heterogeneity in between trials. Mortality also did not significantly differ (OR 1.23, 95% CI 0.76–1.99, *p* = 0.409) while the point estimate was shifted in favor of BMT alone. There was no relevant heterogeneity in between trials.

## Discussion

Over the last 10 years many practice changing trials of endovascular therapy for acute ischemic stroke have been published, beginning with the pivotal trials in large vessel occlusions, followed by extended time window and large core trials 2 years ago [10]. Subgroup analyses of these trials and observational studies suggested a benefit for EVT in more distal occlusions, and also other intracranial vascular territories such as the anterior and posterior cerebral arteries. DISTAL, DISCOUNT and ESCAPE-MEVO showed that EVT did not lead to an additional benefit for patients but there were even trends towards a higher mortality and significantly more symptomatic intracranial hemorrhages. A simple explanation at first sight may just be, that the reduced benefit of less tissue to salvage, higher background efficacy of IVT, a more benign natural course just does not outweigh the possible harm of complications associated with the intervention and everything around it. However, this resembles the situation after publication of the earlier LVO trials that were uniformly negative. Among other possible explanations for consistently negative results throughout these 3 trials, a major issue appears to be a selective and biased recruitment due to a perceived lack of equipoise for EVT in stroke patients with medium and distal vessel occlusions. This is—among others but not exclusively—illustrated by higher age, lower IVT rates mainly because of time window and/or contraindications, lower median NIHSS scores, higher rates of preexisting disability, and poorer than expected outcomes in DMVO when compared to former EVT trials. If we assumed equipoise and unbiased recruitment in future trials, there might be other issues that may lead to disappointing results.

In general, potential therapeutic gain of EVT will likely decrease from proximal to distal, because of lower stroke severity and smaller perfusion deficit in DMVO than in LVO. Inversely, intra- and periinterventional complications may result from a more difficult approach through smaller and more delicate vessels. As a consequence, increased sICH and SAH rates may ruin the positive effects of higher reperfusion rates. Also, with an unbiased recruitment in earlier time windows, the percentage of patients receiving IVT will increase, and IVT becomes more effective in patients with distal occlusions and lower thrombus load, which might again negate any additional effect of EVT. A major drawback of EVT is that the established clinical pathway, i.e. treatment on a stroke unit for older and milder affected stroke patients is often withheld from EVT patients. A significant proportion of the EVT patients will receive endotracheal intubation, and possibly be allocated to an ICU rather than a stroke unit. This increases the risk for complications not directly but indirectly related to EVT, namely higher risks of pneumonia, delirium, ICU complications etc. which by themselves are strong predictors for a poorer prognosis.

Other explanations for the present negative study results also given by the authors pertain to the (mandatory) choice of given devices, approach, and technique applied. This would be a case in point of improved and stricter study protocols with more stringent in- and exclusion criteria. As the more distal vasculature is more tortuous, prone to injury such as perforation, dissection or shearing of small perforating vessels, aspiration may be safer than actual stentretrievers, which were used in ESCAPE MeVO as first device to be applied and 80% of patients in DISTAL (6). In accordance with this, reperfusion rates (around 70–75%) were lower than in previously reported trials especially in LVO patients. Subgroup analyses in one trial suggested a better effect with higher NIHSS scores which was not seen in the other trials, also there are hints towards a more time dependent effect, i.e. the earlier the better. Also a subgroup of patients with favourable perfusion imaging characteristics appeared to fare better with EVT.

At this point we feel that EVT for DMVO is not an established therapeutic option for acute stroke patients until more evidence from randomized trials with adapted protocol designs becomes available. Even more so this holds true, now that ESCAPE MeVO, which did recruit proximal (dominant) M2 occlusions could not show any benefit for these most proximal of the DMVO occlusions. In fact, the point estimate in the subgroup analysis of ESCAPE MeVO was a non-significant adjusted HR of 0.9 (95% CI 0.60–1.33) in favour of BMT over EVT. Now, that there is equipoise for the role of EVT in DMVO including the proximal M2 segment we encourage the international scientific stroke community to rapidly design and institute new trial protocols with a more homogeneous design to allow patient level metaanalyses ensuring the use of the best and safest interventional technique, unbiased recruitment and limitation to for instance the MCA territory with M2 (dominant, codominant, or non-dominant) and at most M3 occlusions, and maybe A1 occlusions only. We feel that P2 and even P1 occlusions, while causing disabling symptoms are difficult for these trials, because high NIHSS scores are rare while persisting hemianopia always will end up with an outcome mRS score of 2, no less or more. It may also be advisable to limit the time window from stroke onset to EVT to less than 6 h and the preexisting disability to mRS scores 0 or 0–1. Advanced imaging to differentiate primary and secondary DMVO might be justified, but will restrict EVT to a few highly selected patients. Finally, protocols contemplating the additional use of postinterventional intraarterial fibrinolytics may be an option.

As noted before, there are major differences in between trial designs including endpoints introducing heterogeneity and limiting at least to a certain extent the validity of our metaanalysis. The results of our analysis therefore need to be interpreted with caution. The primary endpoints differed in between trials, ordinal mRS analysis for DISTAL and ESCAPE MeVO, which was switched in ESCAPE MeVO as defined in the protocol to mRS 0–1 versus 2–6 because of a violation of the proportionality of odds assumption. For DISCOUNT the primary endpoint was mRS 0–2 versus 3–6. As only the mRS score category of 0–2 versus 3–6 was extractable from all three trials we decided to perform the meta-analysis for efficacy on this (secondary) endpoint. Two trials reported their safety outcomes “as treated” only and not “intention to treat”, which may have introduced a bias towards sICH allocation. We neither found a reason for that in the published protocol for ESCAPE MeVO nor the presentation of DISCOUNT. We feel, however, that at least with regard to sICH the results of our analysis show, that there is a high consistency for the respective point estimates.

## Conclusion

Guidelines should at this point clearly state that EVT for DMVO currently is not a standard of care and that these patients should (a) be treated based on individual interdisciplinary decision between stroke neurologist and interventional neuroradiologist and (b) ideally be recruited into rigorously designed clinical trials. To take up again the introductory quote … it´s time to get back up from the ground and try again.

## Data Availability

Not applicable.

## References

[1] Goyal, M., Menon, B. K., van Zwam, W. H., Dippel, D. W., Mitchell, P. J., Demchuk, A. M., et al. (2016). Endovascular thrombectomy after large-vessel ischaemic stroke: A meta-analysis of individual patient data from five randomised trials. *Lancet,**387*(10029), 1723–1731. 10.1016/S0140-6736(16)00163-X 26898852 10.1016/S0140-6736(16)00163-X

[2] Ungerer, M. N., Bartig, D., Richter, D., Krogias, C., Hacke, W., & Gumbinger, C. (2024). The evolution of acute stroke care in Germany from 2019 to 2021: Analysis of nation-wide administrative datasets. *Neurological Research and Practice,**6*(1), 4. 10.1186/s42466-023-00297-x 38200611 10.1186/s42466-023-00297-x PMC10782681

[3] Bosel, J., Hubert, G. J., Jesser, J., Mohlenbruch, M. A., & Ringleb, P. A. (2023). Access to and application of recanalizing therapies for severe acute ischemic stroke caused by large vessel occlusion. *Neurological Research and Practice,**5*(1), 19. 10.1186/s42466-023-00245-9 37198694 10.1186/s42466-023-00245-9 PMC10193718

[4] Turc, G., Bhogal, P., Fischer, U., Khatri, P., Lobotesis, K., Mazighi, M., et al. (2019). European Stroke Organisation (ESO)- European Society for Minimally Invasive Neurological Therapy (ESMINT) guidelines on mechanical thrombectomy in acute ischemic stroke. *Journal of Neurointerventional Surgery,**11*(6), 535–538. 10.1136/neurintsurg-2018-014568 31152058 10.1136/neurintsurg-2018-014568

[5] Goyal, M., Menon, B. K., Krings, T., Patil, S., Qazi, E., McTaggart, R. A., et al. (2016). What constitutes the M1 segment of the middle cerebral artery? *Journal of Neurointerventional Surgery,**8*(12), 1273–1277. 10.1136/neurintsurg-2015-012191 26863104 10.1136/neurintsurg-2015-012191

[6] Beckonert, N. M., Weller, J. M., Alegiani, A. C., Boeckh-Behrens, T., Deb-Chatterji, M., Hamann, G. F., et al. (2024). Endovascular treatment of primary M3 occlusion stroke in clinical practice: Analysis of the German Stroke Registry. *Neurological Research and Practice,**6*(1), 36. 10.1186/s42466-024-00330-7 39020409 10.1186/s42466-024-00330-7 PMC11256396

[7] Sembill, J. A., Sprugel, M. I., Haupenthal, D., Kremer, S., Knott, M., Muhlen, I., et al. (2024). Endovascular thrombectomy in patients with anterior circulation stroke: An emulated real-world comparison. *Neurological Research and Practice,**6*(1), 37. 10.1186/s42466-024-00331-6 39049127 10.1186/s42466-024-00331-6 PMC11270839

[8] Psychogios, M., Brehm, A., Ribo, M., Rizzo, F., Strbian, D., Raty, S., et al. (2025). Endovascular treatment for stroke due to occlusion of medium or distal vessels. *New England Journal of Medicine,**392*(14), 1374–1384. 10.1056/NEJMoa2408954 39908430 10.1056/NEJMoa2408954

[9] Goyal, M., Ospel, J. M., Ganesh, A., Dowlatshahi, D., Volders, D., Mohlenbruch, M. A., et al. (2025). Endovascular treatment of stroke due to medium-vessel occlusion. *New England Journal of Medicine,**392*(14), 1385–1395. 10.1056/NEJMoa2411668 39908448 10.1056/NEJMoa2411668

[10] Mocco, J. (2025). Medium- and distal-vessel occlusion - the limit of thrombectomy? *New England Journal of Medicine,**392*(14), 1440–1442. 10.1056/NEJMe2500492 39908428 10.1056/NEJMe2500492

